# Interferometric measurements of many-body topological invariants using mobile impurities

**DOI:** 10.1038/ncomms11994

**Published:** 2016-06-17

**Authors:** F. Grusdt, N. Y. Yao, D. Abanin, M. Fleischhauer, E. Demler

**Affiliations:** 1 Department of Physics and Research Center OPTIMAS, University of Kaiserslautern, Kaiserslautern 67663, Germany; 2 Graduate School Materials Science in Mainz, Gottlieb-Daimler-Strasse 47, 67663 Kaiserslautern, Germany; 3 Department of Physics, Harvard University, Cambridge, Massachusetts 02138, USA; 4 Department of Physics, University of California, Berkeley, California 94720, USA; 5 Department of Theoretical Physics, University of Geneva, 24 quai Ernest-Ansermet, 1207 Geneva, Switzerland

## Abstract

Topological quantum phases cannot be characterized by Ginzburg–Landau type order parameters, and are instead described by non-local topological invariants. Experimental platforms capable of realizing such exotic states now include synthetic many-body systems such as ultracold atoms or photons. Unique tools available in these systems enable a new characterization of strongly correlated many-body states. Here we propose a general scheme for detecting topological order using interferometric measurements of elementary excitations. The key ingredient is the use of mobile impurities that bind to quasiparticles of a host many-body system. Specifically, we show how fractional charges can be probed in the bulk of fractional quantum Hall systems. We demonstrate that combining Ramsey interference with Bloch oscillations can be used to measure Chern numbers characterizing the dispersion of individual quasiparticles, which gives a direct probe of their fractional charges. Possible extensions of our method to other many-body systems, such as spin liquids, are conceivable.

## Abstract (web summary)

Probing topological orders in many-body systems remains a major challenge, which requires bulk-edge correspondence. Here, Grusdt *et al.* present an approach to show that fractional charges can be directly probed in the bulk of fractional quantum Hall systems using mobile impurities through interferometric measurements.

## Graphical abstract

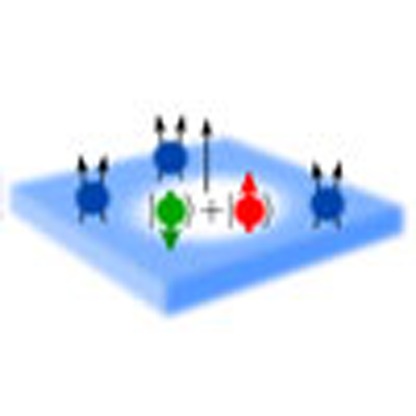

Many-body systems with spontaneous symmetry breaking can be described by Ginzburg–Landau theories, formulated in terms of local order parameters. This powerful approach provides a universal description of systems with very different microscopic Hamiltonians but with similar type of symmetry breaking, such as superfluids and ferromagnets. The integer and fractional quantum Hall effects^1^,^2^,^3^ in contrast are examples of quantum phases of matter, for which no local order parameters exist. Instead, these systems are described by non-local topological invariants^4^. The fractional charges of elementary excitations^2^,^3^, the many-body Chern number 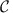 (ref. 5) and, in the case of quantum spin liquids, fractional quantum Hall systems and fractional Chern insulators^6^,^7^,^8^,^9^,^10^,^11^,^12^,^13^, the groundstate degeneracy on a torus^14^, constitute important examples of topological order parameters.

Probing—and, in some cases, even defining—the non-local order parameters of topological systems with strong correlations represents a major experimental and theoretical challenge. Many indicators used in the theoretical description of such systems, such as the entanglement entropy^15^,^16^ and spectrum^17^, are difficult to probe directly in current experiments, although first steps in this direction have been undertaken^18^,^19^,^20^,^21^. Previously, it has been shown that edge excitations can be used to detect topological orders by measuring their fractional charges^4^,^22^,^23^,^24^ and statistics^4^.

Motivated by the coherent control of mesoscopic quantum systems achievable in recent experiments, we explore new ways of detecting topological order. Our method is ideally suited to systems of ultracold atoms, which recently emerged as a new promising platform for realizing and probing various topological states of matter. The ability to perform interferometric measurements in such systems is one of their key technical advantages in comparison with other experimental set-ups. Cold atoms provide a versatile toolbox, allowing to engineer not only single-particle properties of Hamiltonians, such as the shape of optical lattice potentials, but also the interactions between particles^25^,^26^,^27^,^28^. Recently, the Chern number has been measured in transport experiments^29^ and the celebrated Haldane model has been realized^30^ in systems of weakly interacting ultracold atoms. An experimental realization of the fractional quantum Hall effect in such systems^6^,^7^,^31^,^32^,^33^,^34^,^35^,^36^ should be within reach with the currently available tools. In addition, direct and fully coherent control over individual atoms has been demonstrated in experiments with ultracold quantum gases, see, for example, refs 37, 38.

Here we present a concept of using impurity atoms as coherent probes of the topological invariants of strongly correlated many-body systems of host atoms. Our approach allows one to measure topological order parameters directly in the bulk of the system, without the need of relying on the bulk-edge correspondence. The main idea is to map out the topology characterizing the effective bandstructure of elementary quasiparticle (qp) excitations. As will be pointed out, it is intimately related to the topological order of the groundstate. In particular, we show how the Chern numbers of the effective qp bandstructures can be measured by combining Bloch oscillations with a Ramsey interferometric sequence. We point out that they are directly related to the corresponding (fractional) charges for arbitrary Abelian quantum Hall states, and show how the Chern number of the many-body groundstate can be derived. Our scheme extends earlier ideas^39^,^40^,^41^,^42^, which have been developed to measure topological invariants of essentially non-interacting particles (ultracold atoms in particular), to the realm of strongly correlated quantum many-body systems. More generally, our interferometric method paves the way for a detailed investigation of qp properties, including, possibly, their braiding statistics.

## Results

### Interferometric detection of many-body Chern numbers

The key idea of our approach is to measure the Chern number of the effective qp bandstructure using a generalization of the interferometric technique developed for non-interacting systems in refs 39, 40. First, let us briefly summarize the main idea of the interferometric protocol for a weakly interacting Bose–Einstein condensate loaded in a two-dimensional (2D) Bloch band in a system with an effective magnetic field. Because of their experimental relevance for ultracold atoms we will discuss lattice systems. The first ingredient to measure the corresponding Chern number 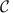 is a direct detection of the geometric Zak phase 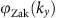
39 for a given value of the quasimomentum *k*_*y*_.

**Figure 1:**
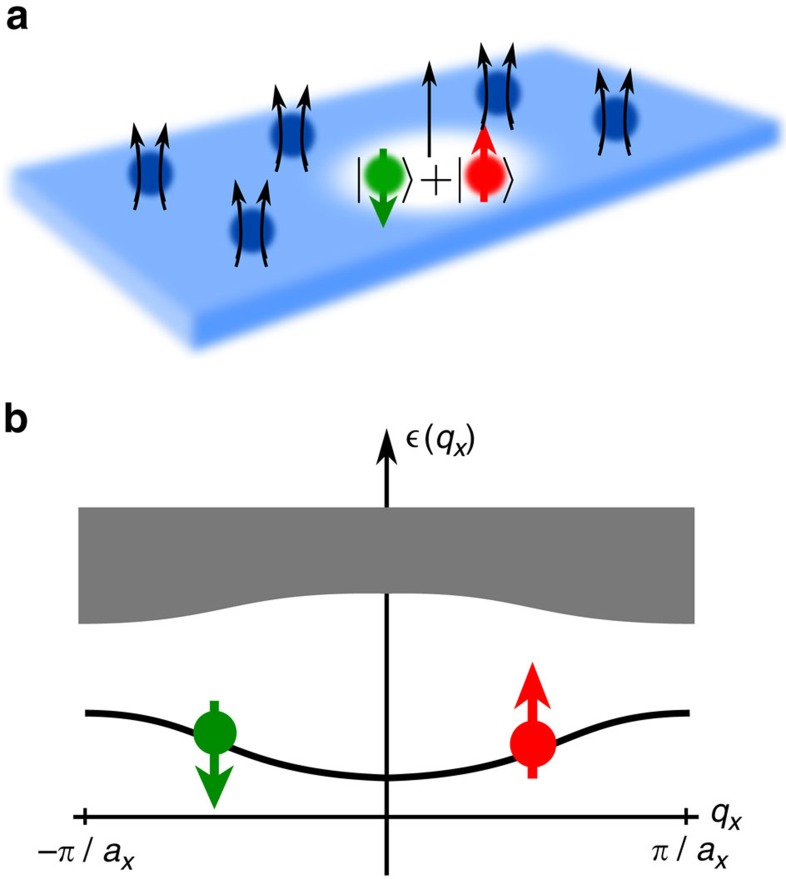
Topological polarons. We propose a scheme for the measurement of many-body topological invariants of interacting states with topological order. It can be applied to measure the Chern numbers of Abelian quantum Hall states (for example, of Laughlin type) and of their topological excitations, as illustrated in **a**. An elementary excitation (here a quasihole) is coupled to a mobile two-component impurity. When the impurity is tightly bound to the quasihole, a topological polaron is formed. It can be labelled by its quasimomentum *q*, and its two internal degrees of freedom ↑, ↓ and can be used to perform interferometry. The spectrum 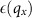 of the many-body system is depicted in **b** for a generic one-dimensional case. The topological invariant of the qp bandstructure can be measured using tools developed for non-interacting systems by a combination of Bloch oscillations and Ramsey interferometry.

To this end, the condensate is moved to (*k*_*x*_, *k*_*y*_), where a *π*/2 Ramsey pulse is used to prepare a superposition of two internal states *σ*=↑, ↓. The initial wavefunction thus reads 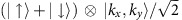. Next, sufficiently weak opposite forces **F**(↓)=−**F**(↑) are applied such that the two components undergo Bloch oscillations (Fig. 1b). When the force is applied along the *x* axis for a time Δ*t*=*G*_*x*_/2*F*, where *G*_*x*_ denotes the width of the magnetic Brillouin zone (BZ) containing an integer number of magnetic flux quanta, the two components pick up a relative geometric phase 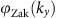 (ref. 43) and the wavefunction reads 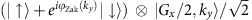. By recombining the two states using a second *π*/2 Ramsey pulse, the Zak phase can be read out. This measurement can be repeated for different values of *k*_*y*_ (ref. 40), and the winding of the Zak phase across the magnetic BZ in the *k*_*y*_ direction (size *G*_*y*_) gives the Chern number^44^,


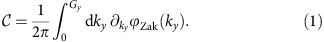


For more details, including the discussion of dynamical phases and gauge dependence of the Zak phase, the reader is referred to refs 39, 40, 41, 42.

Now we extend the interferometric protocol to strongly correlated systems. The key idea is to apply the exact same sequence as described above to a single qp excitation in the host system. To obtain fully coherent control of the qp, it is coupled to a mobile impurity. The resulting composite object (the impurity bound to the topological excitation) will be called a topological polaron (TP), which is at the heart of our scheme. (We call this bound state a TP because, as will be shown, it inherits the topological properties of the qp excitation.) We assume that the impurity has an internal degree of freedom *σ*, the only role of which is to enable Ramsey interferometry as described above. By introducing two pseudospin components *σ*=↑, ↓ a measurement of their relative phase is sufficient, that is, one of the pseudospin states effectively acts as a reference for the second. By applying opposite forces *σ*^*z*^*F***e**_*x*_ directly to the two impurity components for the same time Δ*t*=*G*_*x*_/2*F* as in the non-interacting case (where *G*_*x*_ is defined for host particles), a topological invariant can be measured, which will be identified as the Chern number 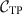 of the effective TP bandstructure (for more details see Methods section). Note that now |*k*_*x*_, *k*_*y*_〉 stands for the full many-body wavefunction describing the TP at quasimomentum (*k*_*x*_, *k*_*y*_), and in this sense true many-body Zak phases 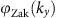 are measured.

Now our main results are summarized. We will establish that the Chern number of the TP bandstructure is directly related to the fractional charges of the qps. We will use Chern–Simons effective field theory, valid at low energies, for the description of Abelian fractional quantum Hall states and their excitations. In addition, we introduce a numerically exact technique for calculating Zak phases of TPs for small lattice systems relevant for experiments. As a concrete example, Laughlin-type fractional Chern insulator states at filling fraction *ν*=1/*m* (defined as a ratio of particle density to flux density) will be considered. For that case, we show that the TP Chern number, discussed in the protocol above, is given by the inverse of the fractional qp charge e*=*e*/*m* (*e* is the charge of particles in the host many-body system),


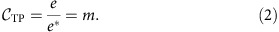


Therefore, a measurement of the TP Chern number directly yields the fractional qp charge.

The result in equation (2), moreover, indicates a direct relation to the many-body Chern number 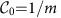 (ref. 5) of the incompressible *ν*=1/*m* Laughlin groundstate, 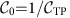. (By a fractional Chern number 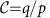, with *q*, *p* integers, we refer to a situation where *p* degenerate states on a torus share a total Chern number *q*, giving rise to a fractionally quantized Hall conductivity 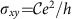 (ref. 5). *q* is an integer topological invariant characterizing the vector bundle defined by the set of degenerate states on a torus.) Similar relations are true for any Abelian quantum Hall state, where *e*/e* needs to be expressed in terms of the *K*-matrix. In that case there exist *J*=1, ..., *n* different qp types, with fractional charges 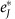 and qp Chern numbers 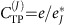. The Chern number of the incompressible fractional quantum Hall state is given by 
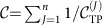
 (see Methods section).

### TPs and qp Chern numbers

Our discussion of TPs will be organized as follows. In this section we focus on the physical picture of TPs in fractional quantum Hall systems. We discuss the origin of equation (2), assuming that an impurity particle is strongly bound to a qp. Therefore, the impurity current 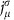 (where *μ*=*x*, *y*, *t* and *σ*=↑, ↓) is equivalent to the qp current, which is conserved because of a global *U*(1) symmetry. Detailed discussion of the binding mechanism between the impurity and a qp will be presented in the next subsection.

As in the theoretical proposal of ref. 40 and the experimental demonstration of ref. 39, we assume that the two-component TP can be controlled by the force **F** acting on the impurity. (In experiments a spin-dependent force **F** could be realized by a magnetic field gradient.) We model this by coupling the TP current 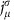 to an external field that acts differently on the two internal states of the impurity but does not affect host particles. We describe this field by the potential *B*_*μ*_(*σ*) so that the external force acting on the impurity is given by *F*_*i*_(*σ*)=−*q*(∂_*t*_*B*_*i*_+∂_*i*_*B*_0_), where *q* denotes the charge of the impurity associated with the field *B*_*μ*_. We emphasize that the impurity is not affected directly by the gauge field creating a fractional quantum Hall state for host particles. However, by binding a qp of the surrounding fractional quantum Hall state it acquires topologically non-trivial dynamics. This can be understood as arising from the particle–vortex duality of the fractional quantum Hall effect, which implies that the moving qp bound to the impurity sees surrounding host particles as a source of effective flux.

To avoid complications arising because of an underlying lattice, we first employ an effective field theory to capture the universal topological properties of our system (in particular, Chern numbers). It is valid when all relevant length scales arising from gauge fields and interactions are large compared with the lattice constant. Using this Chern–Simons description of the fractional quantum Hall effect one can show (see Methods section for details) that the effective Lagrangian describing the TP is given by (throughout the paper we set *ħ*=1)


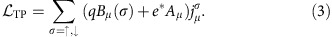


Here *A*_*μ*_ denotes the external gauge field corresponding to the homogeneous magnetic field *b*_*z*_ seen by the host particles in the many-body quantum Hall system, ∂_*x*_*A*_*y*_−∂_*y*_*A*_*x*_=*b*_*z*_. In the case of ultracold atoms, *b*_*z*_ corresponds to an artificial gauge field^45^. The second term in the Lagrangian (3) describes the emergent coupling of the TP to the gauge field *A*_*μ*_, albeit with a fractional *A*_*μ*_ charge e*=*e*/*m*. Hence, according to equation (3) the TP sees an effective magnetic field 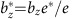. Note that we consider a situation where the host system has a global *U*(1) symmetry, giving rise to conserved charges (or particle numbers in the context of ultracold atoms).

We emphasize again that the emergent coupling of impurity particles to the gauge field *A*_*μ*_ arises only through binding of a qp excitation. For Laughlin states, a qp acquires an Aharonov–Bohm phase 2*π* when going around a single host particle. Hence, the effective flux density seen by the qp is equal to the density of host particles, which is 1/*m* times the magnetic flux density of *A*_*μ*_.

Now the Chern number 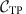 of the TP can easily be calculated. To this end we note that, in a homogeneous magnetic field *b*_0_, the Berry curvature 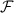 is constant, 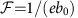. Similar to the manner in which we described the interferometric protocol above, we defined the TP Chern number by integrating the Berry curvature over the entire magnetic BZ of the host many-body system. Its size is *G*_*x*_ × *G*_*y*_=2*πeb*_*z*_, where *b*_*z*_ is the magnetic field seen by host particles. Because the Berry curvature seen by the TP is 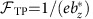 we obtain


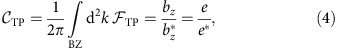


as claimed in equation (2).

Note that because of gauge invariance 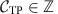 has to be an integer^46^,^47^, as long as the TP groundstate is non-degenerate for all *k* (see also Methods section). Therefore, when the qp charge is not the inverse of an integer, the band of TP groundstates needs to have degeneracies. When e*/*e*=*p*/*q*, where *p* and *q* are relative prime, we expect *p* degenerate bands sharing a total Chern number of 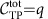. We find numerical signatures for this degeneracy, and a specific example is discussed in the Supplementary Note 1.

Alternatively, the qp charge e*/*e* of the TP could be obtained directly from an interferometric measurement of the Aharonov–Bohm phase 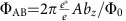 in the magnetic field *b*_*z*_. Here *A* denotes the area encircled by the TP and Φ_0_ is the magnetic flux quantum. Because obtaining control over the impurity momentum is technically less challenging than achieving full spatial resolution, the interferometric measurement of the Chern number should be easier to implement.

### Microscopic description of TPs

Now we provide a microscopic description of TPs in correlated many-body systems interacting with a single impurity particle. To this end, we investigate concrete models of interacting fermions on a lattice, whose groundstates are integer and fractional Chern insulators^6^,^7^,^8^,^9^,^10^,^11^,^12^. We develop an exact numerical method to solve this problem and compare our results to an approximate strong coupling theory of TPs, which we also introduce below. Like before, we concentrate on states in the Laughlin universality class, with a single qp type. Although the field theory employed so far assumed a continuous system in a homogeneous magnetic field, it should describe correctly the universal topological properties of the lattice systems, which will be investigated below.

We consider a topologically ordered phase in a many-body system (referred to as the host system), described by the groundstate of a Hamiltonian 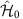. For probing qp excitations, we introduce a mobile impurity with two internal states, described by 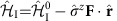. Here 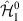 denotes the kinetic energy and 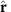 the position operator of the impurity. The two internal states ↑ and ↓ of the impurity experience opposite forces ±**F**, as described by the Pauli matrix 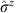. To bind qps to the impurity, a local interaction 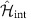 with the many-body system is introduced. Its concrete form can differ from model to model; however, for simplicity we will assume throughout that it is independent of the internal state of the impurity. Thus, our system is described by the Hamiltonian 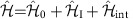. Local interactions between particles with multiple internal states are routinely realized in systems of ultracold atoms.

In equilibrium, that is, for **F**=0, the groundstate 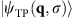 describes a TP and can be labelled by its quasimomentum **q** and its internal state *σ*=↑, ↓. The external force **F** couples to the quasimomentum **q** of the TP. This drives Bloch oscillations where the quasimomentum changes according to 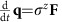, where *σ*^*z*^=1 (*σ*^*z*^=−1) for *σ*=↑ (*σ*=↓), respectively). By applying the scheme described in ref. 39 to the states 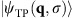 the geometric Zak phases characterizing the TP bandstructure can be measured (Fig. 1). As discussed in the Methods section, the corresponding Chern number is obtained by integrating the Berry curvature seen by the TP over the magnetic BZ of the host many-body system.

### Strong coupling theory

Before turning our attention to concrete models, we introduce the strong coupling theory of TPs, which is inspired by Landau's and Pekar's treatment of the polaron problem in polar crystals^48^,^49^. There are several important energy scales for describing TPs. First, qp excitations are characterized by the bandwidth *J*_qp_ of their effective dispersion and by the energy required for their creation, which is set by the bulk excitation gap Δ_0_. Second, the impurity is characterized by the effective hopping *J*_I_ and the coupling strength to the host particles *V*. Our description of TPs requires the following hierarchy of scales. The qp gap Δ_0_, which is larger than *J*_qp_, should be larger than *J*_I_ and *V*, that is, 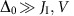. The impurity–host particle interaction strength *V* should be chosen such that the impurity binds precisely one qp. We also need *J*_qp_ to be smaller than *J*_I_, that is, 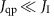 so that the total momentum of the TP, which is effectively shared by the impurity and the qp, resides predominantly in the qp. Another way of understanding this requirement is that the impurity should be fast compared with the qp and thus follow its dynamics adiabatically.

Next, we introduce a frame where the total momentum of the TP is conserved explicitly. To this end, we restrict ourselves to a single qp and approximate 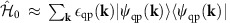, with 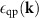 being the effective dispersion of qps and 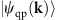 the qp state with momentum **k**. We apply the unitary transformation 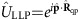 introduced by Lee, Low and Pines (LLP)^50^, where 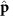 is the impurity momentum operator and 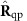 is qp position operator conjugate to its momentum operator 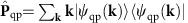. The transformed Hamiltonian 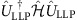 reads


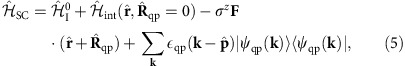


as will be explained now.

The kinetic part of the impurity Hamiltonian commutes with the impurity momentum, 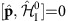, and remains unchanged. 
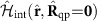
 denotes the interaction Hamiltonian for a qp localized in the origin of the new polaron frame, that is, 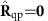. Here we assumed that (within the single qp approximation) 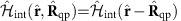 depends only on the relative distance between impurity and qp, and we used that 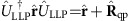. Finally, because 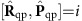 the qp momentum is shifted by an amount 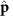 in the last line, 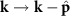.

Under the strong coupling conditions outlined above, we can make a product ansatz for the TP wavefunction, 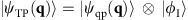, where the impurity follows the qp adiabatically. In the polaron frame the impurity sees a quasistatic potential created by the qp and its wavefunction 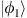 is determined by the strong coupling impurity Hamiltonian


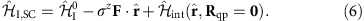


This leads to a modification of the effective qp dispersion in equation (5), which we approximate by 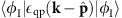. We proceed by eliminating the last term in the first line of equation (5) by a time-dependent gauge transformation 
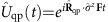
. In the resulting effective Hamiltonian the force **F** couples to the conserved momentum **k** and the qp Hamiltonian in the strong coupling theory thus reads


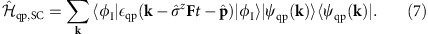


Next, we discuss how the topological invariant measured by the TP relates to the Chern number of the effective qp bandstructure using the strong coupling wavefunction. During the protocol described above (see also Fig. 1) the force 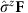 is applied and the TP wavefunction 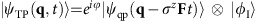 follows its groundstate adiabatically. Thereby, it picks up a phase 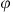 containing a geometric part of 2*πν*_TP_, which is measured by the Ramsey sequence (dynamical phases are discussed in refs 39, 40). Owing to the product form of the strong coupling wavefunction we find two contributions, *ν*_TP_=*ν*_qp_+*ν*_I_.

The first contribution is picked up by the qp wavefunction, 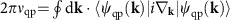. When the path in momentum space described by the TP in the interferometer encloses the (magnetic) BZ, the qp invariant is related to the TP Chern number defined above,


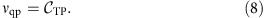


The second contribution *ν*_I_ is picked up by the impurity part of the wavefunction. In the adiabatic limit of small **F** it is 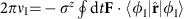. This term corresponds to a geometric phase because it does not vanish in the limit when **F**→0 and needs to be considered in general. It measures the displacement of the impurity wavefunction relative to the qp located in the origin of the polaron frame (recall that 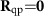 in the interaction Hamiltonian) and can become relevant in lattice systems. However, when a closed loop 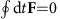 is considered as in the interferometric sequence we discuss, the impurity invariant vanishes, *ν*_I_=0.

### TPs in Chern insulators

Now we turn our attention to a concrete model of interacting particles on a lattice, described by the Hofstadter–Hubbard Hamiltonian


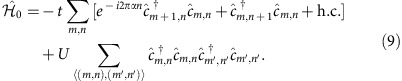


The first term is the celebrated Hofstadter model^51^, and it describes free particles hopping between the sites (*m*, *n*) of a square lattice in a magnetic field (using Landau gauge), where *α* denotes the magnetic flux density per plaquette (in units of the flux quantum) and *t* is the hopping amplitude. The second term describes nearest neighbour interactions of strength *U* between the particles. Here we consider fermions for concreteness, 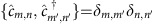, but a similar Hofstadter–Hubbard model has also been discussed for bosons with contact interactions^6^,^7^. For sufficiently small values of *α*, the groundstates of equation (9) show the *integer and fractional quantum Hall effect* depending on the filling fraction *ν*.

To study TPs we consider the Hofstadter–Hubbard model equation (9) at filling *ν*=1/*m* on a torus. We choose the number of flux quanta in the host many-body system *N*_φ_=*Nm*+1 such that the groundstate of 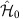 contains one quasihole (qh) excitation. Next, we add a single impurity, described by 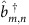, hopping between the sites (located at positions **r**_*m*,*n*_) of the same 2D lattice,


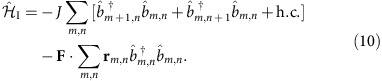


To bind the impurity to the qh, its interaction with the surrounding fermions is modelled by a repulsive contact potential, 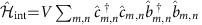. In the following we consider an impurity with only a single internal state for simplicity.

The model proposed above (equations (9) and (10)) can be implemented with ultracold atoms. In refs 52, 53 the Hofstadter Hamiltonian was realized for bosons, and interactions can be introduced by using deep optical lattices. The impurity could be realized by adding a second atomic species, with different meta-stable internal states.

In the Methods section we present a formalism for calculating the full many-body TP wavefunction 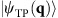 exactly for a given total momentum **q**, based on the LLP polaron transformation. Here we use this approach to obtain both the dispersion relation and the Chern number of TPs.

**Figure 2:**
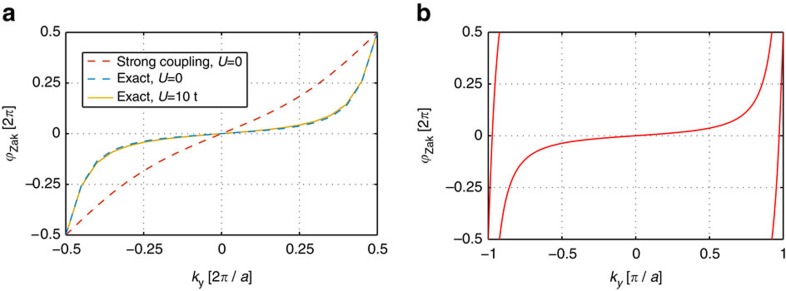
Many-body Chern numbers. A single mobile impurity can be coupled to an elementary hole excitation of an integer (**a**) or fractional (**b**) Chern insulator, and form a TP. The winding of the many-body Zak phase 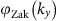 across the BZ defines the many-body Chern number of the TP. In **a** we compare predictions for integer Chern insulators without (*U*=0) and with interfermion interactions (*U*≠0). Parameters are *J*=*t*/2, *V*=2*t*, *α*=1/4 and we simulated 4 × 4 sites with *N*=3 fermions. In **b**, a *ν*=1/3 fractional Chern insulator is considered, for parameters *J*=*t*/2, *V*=2*t*, *α*=1/4, *U*=10*t*. We simulated 4 × 7 sites filled with *N*=2 fermions, corresponding to 3*N*+1=7 flux quanta as required for having one quasihole excitation.

In Fig. 2a our results are shown for an integer Chern insulator, with and without fermion–fermion interactions *U*. We consider the case *α*=1/4 and use Landau gauge where the size of the magnetic unit cell is (*a*_*x*_=4*a*) × *a*, with *a* denoting the lattice constant. We calculate the Chern number of the TP from the winding of the many-body Zak phase, 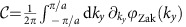
44, see Methods section for details. For comparison, the result of a simple strong coupling analysis is shown, where the impurity is bound to a free hole. Although the distribution of Berry curvature differs, the strong coupling theory predicts correctly the TP Chern number, 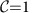.

In Fig. 2b we repeat our calculations for a fractional Chern insulator at *ν*=1/3, corresponding to a 1/3 Laughlin-type state. (We checked numerically that the incompressible groundstate of 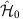 has a fractional Chern number 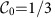 and the expected threefold groundstate degeneracy on a torus.) The Chern number of the TP, which is determined as the winding of the Zak phase of the TP in Fig. 2b, has the value 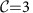. This is expected from the field-theoretical arguments given in the beginning (equation (2)).

An important quantity for estimating the robustness of our protocol is the energy gap of the TP state. To get some analytical understanding, we first consider the continuum limit *α*→0 when the system enters the quantum Hall regime. Here the impurity (mass *M*_I_) interacts with fermions (mass *M*_F_) in a filling-*ν* Laughlin state through a local contact interaction 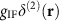. Within the strong coupling theory introduced above, we find a finite TP-binding energy given by


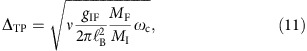


where 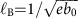 is the magnetic length and 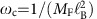 the cyclotron frequency.

**Figure 3:**
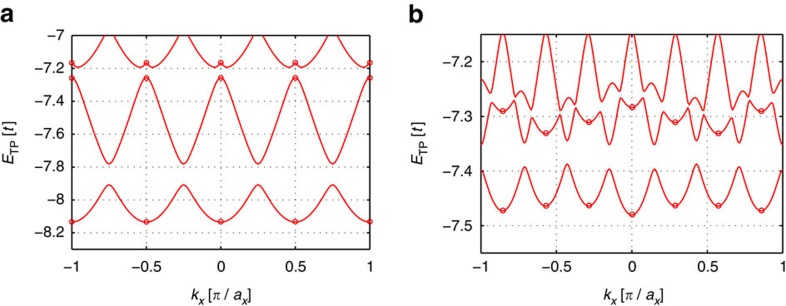
TP spectrum. The energies of the lowest TP eigenstates are shown for *k*_*y*_=0 as a function of the lattice moment *k*_*x*_, calculated in the polaron frame (see Methods section). In **a** an integer Chern insulator is considered for *U*=10*t*, *J*=*t*/2, *V*=2*t*, *α*=1/4 and we simulated 4 × 4 sites with *N*=3 fermions. In **b** the case of a fractional Chern insulator is shown, for parameters *J*=*t*/2, *V*=2*t*, *α*=1/4, *U*=10*t*. We simulated 4 × 7 sites filled with *N*=2 fermions. Circles mark the positions of the discrete momenta defined by the finite quantization volume.

In Fig. 3 we calculate the TP spectra numerically for small lattice systems and find low-energy states where the impurity is bound to the qh forming the TP. We observe that the TP gap Δ_TP_ shows large finite-size effects. The TP-binding energy oscillates at a frequency given by the system size, but at the discrete momenta allowed by the finite quantization volume—which are indicated in the figures by circles—we find the largest values of the TP gap. In the case of integer Chern insulators, this TP gap constitutes a sizable fraction of the cyclotron gap 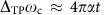. Note that *ω*_c_ provides an upper bound for the TP gap, Δ_TP_<*ω*_c_, because at this energy unbound excitons can be created in the bulk. In the case of fractional Chern insulators in contrast, the TP gap is only a small fraction of the cyclotron energy, but so is the bulk gap Δ_LN_ of the fractional Chern insulator, Δ_LN_<*ω*_c_ (ref. 7). In this case Δ_LN_ similarly provides an upper bound for the TP gap as the cyclotron energy in the integer Chern insulator case, Δ_TP_<Δ_LN_.

To form a TP for mapping out the topological order of the host many-body system, we wish to realize the strong coupling limit described above. Thus, for impurity dynamics to be fast compared with qp tunnelling, a weak qp dispersion is desirable. This can be achieved in lattice systems by including long-range hoppings for the host particles, see ref. 54. We simulated TPs in systems with long-range tunnellings of this kind (see also Supplementary Note 1) and verified that the TP Chern number 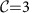 is a robust feature of the groundstates.

## Discussion

By coupling a mobile impurity to the topological qp excitations of an incompressible many-body groundstate of host particles, a TP can be formed. Using internal degrees of freedom of the impurity, fully coherent control can be gained over individual qp excitations of the host many-body system. We demonstrated that TPs can be used to measure the topological invariants characterizing such qp excitations. In particular, we developed an interferometric measurement scheme for qp Chern numbers and showed that these are directly related to the fractional charges of topological excitations in two dimensions. To this end, we generalized schemes developed earlier for non-interacting systems and showed by explicit calculations that our scheme can be applied to integer- and fractional quantum Hall systems and Chern insulators.

In systems of ultracold atoms, impurities can be realized, for example, by introducing another atomic species and their internal degree of freedom corresponds to different hyperfine states. To neglect the effects of interactions between TPs, the density of impurity atoms needs to be well below one atom per cyclotron orbit. We expect that, in order to realize TPs in an experiment, a main challenge will be their preparation. Because TPs can carry fractional charges as in the case of fractional Chern insulators, their preparation requires non-local operations in general. Therefore, we suggest to build impurity atoms into the state as defects already before preparing the incompressible many-body state of host atoms. One concrete approach would be to start from this system and cool it down into its groundstate. If the densities of majority and impurity atoms are suitably chosen, this leads to the formation of TPs. Alternative approaches have also been discussed in the literature^55^,^56^,^57^,^58^.

The method presented in this article can provide a powerful experimental tool for detecting topological order in interacting many-body systems. It is ideally suited for cold atom experiments, which offer a rich toolbox with precise and fully coherent control over individual atoms. The concept of TPs as experimental probes for topological order can be generalized to other systems, however, including, for example, qp excitations on topological superconductors, or systems with symmetry-protected topological orders. Another interesting direction would be to probe the (non-Abelian) braiding statistics of anyons by coupling them to impurities and forming TPs. In this case, one can envision interferometric sequences designed to probe braiding statistics, which work in real-space rather than momentum space, as considered in this work.

We expect that our idea—which is to witness topological order in a groundstate by probing the topology characterizing the effective bandstructure of its qp excitations—may also be of broader theoretical interest. It provides a direct route how the concepts developed for non-interacting systems can be generalized to correlated many-body systems: we can define a set of topological invariants for a many-body state by considering single-particle topological invariants of all qp excitations. These may include Chern numbers, 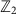 invariants or quantized Zak phases. The approach may be useful, for example, in the study of fractional topological insulators^59^ or quantum spin liquid states^14^. In the case of chiral quantum spin liquids the analogy to our work is particularly close. On a mean-field level the chiral quantum spin liquid can be described by an integer Chern insulator formed by spinons with a unity Chern number (for example, ref. 60). Therefore, it would be interesting to study the binding of a spinon to a mobile impurity and apply the interferometric method introduced in our paper to detect the spinon Chern number.

## Methods

### Chern number of TPs

In the main text we generalized the interferometric protocol developed for the measurement of Chern numbers of non-interacting particles^39^,^40^ to a single qp excitation in a strongly correlated many-body system. The coupling to an impurity particle was necessary for adapting the interferometric protocol. In this way a topological invariant of the TP was defined which, as we will now argue, is the Chern number of the TP.

Consider a qp state 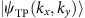, which is characterized by its quasimomentum (*k*_*x*_, *k*_*y*_). Let us assume that the state is non-degenerate for all quasimomenta. The corresponding Chern number 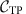 is then defined by the quantized Hall response to an external force **F**. In the case of TPs the external force **F** acts directly on the impurity and couples to the quasimomentum according to 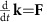. Using the Kubo formula, Thouless *et al.*^46^ have shown that the Chern number can be defined as an integral of the TP Berry curvature 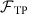 over the (magnetic) BZ,


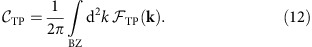


The Berry curvature 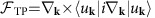 is defined through the Bloch wavefunction 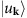 constructed from 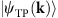. The magnetic BZ is defined by the periodicity of the qp Hamiltonian, including gauge-dependent vector potentials^61^. The periodicity of the TP wavefunction 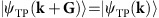, where **G** is a reciprocal lattice vector, guarantees the integer quantization of the Chern number^46^,^47^.

The magnetic BZ of the TP is determined by the microscopic details of the model for both the impurity as well as the host many-body system. Let us assume that the impurity either lives in the continuum (such that effectively the impurity lattice constant *a*→0 vanishes) or that the unit cell of the impurity lattice fits into the magnetic unit cell of the host many-body system an integer number of times in a commensurable way. In either case, the magnetic BZ of the TP is then equal to the magnetic BZ of the host many-body system. This explains why, in our interferometric protocol, the TP Chern number needs to be defined as the winding of the Zak phase of the TP 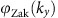 over the magnetic BZ of the host many-body system, *k*_*y*_→*k*_*y*_+*G*_*y*_.

### Field theory of TPs

Here we discuss a field theory description of TPs, allowing us to derive the topological invariants characterizing their effective bandstructure. We consider a more general situation than discussed in the main text, and allow quantum Hall states with an arbitrary Abelian topological order. The generalization of our interferometric protocol to this case is discussed in the end.

Our starting point is a field-theoretical description of the topologically ordered host many-body system. We consider an incompressible groundstate with an Abelian topological order, described by a Chern–Simons theory of level *n*. Such theories are believed to classify all Abelian topological orders and are relevant, for example, for the hierarchical description of the fractional quantum Hall effect^62^ or multilayer fractional quantum Hall systems. They are characterized by the symmetric integer *n*-by-*n* matrix 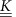 and the charge vector 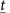. The Lagrangian is^4^


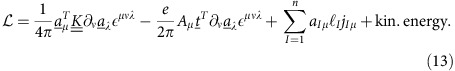


Here 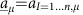 are the auxiliary compact *U*(1) gauge fields from which the conserved current *J*_*μ*_ of the many-body system can be derived, 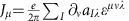. As usual, *μ*, *ν*, ...=*t*, *x*, *y* denote temporal and spatial coordinates.

The first two terms of the Lagrangian equation (13) describe the response of the many-body system to the external *U*(1) gauge field *A*_*μ*_. Here *e* denotes the *A*_*μ*_ charge of the indistinguishable host particles constituting the many-body system. From the Euler–Lagrange equations the quantized Hall response is obtained, 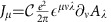, where the many-body Chern number is given by


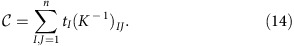


The third term in equation (13) describes the conserved currents *j*_*Iμ*_ of the *I*=1...*n* different qps (in the bulk of the system). The integers 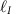 denote the number of qps that are bound together, in particular, 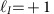 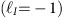 for elementary qp (qh) excitations. Edge terms and kinetic energy corrections will be ignored in the Lagrangian equation (13) in the following. The qps carry fractional charges 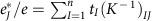 (ref. 4). Thus, we note that the Chern number of the incompressible groundstate is given by the sum of the fractional charges of elementary topological excitations, 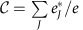.

When the impurity (in a given internal state) binds 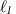 qps of type *I*, their currents can be directly related to the TP current *j*_*μ*_ by 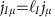. This is demonstrated by a microscopic calculation in the main text. Using this expression and integrating out the auxiliary *U*(1) gauge fields 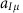 in the Lagrangian equation (13) (ref. 63), we obtain


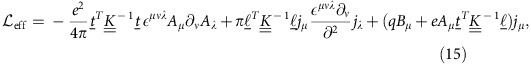


where 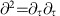. We also included a coupling *qB*_*μ*_*j*_*μ*_ of the impurity to an additional external field *B*_*μ*_. The first term in equation (15) is a Chern–Simons term for the external gauge field *A*_*μ*_. The second term describes the braiding statistics of the TP, which coincides with the expected qp statistics, see, for example, ref. 64. The statistical phase *e*^*iθ*^ picked up when interchanging two TPs adiabatically is given by 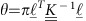 (ref. 4). The last term, most important to our discussion, corresponds to an effective gauge field 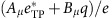 seen by the TP, where the *A*_*μ*_ charge of the TP is given by 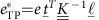.

By applying the interferometric protocol introduced in the main text to different flavours *J*=1, ..., *n* of TPs, all the Chern numbers (or, equivalently, all the fractional charges)


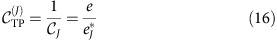


of elementary topological excitations can be measured. To this end a single qp (or qh) of flavour *J* is bound to the impurity. The last equation is then derived as equation (4) in the main text. When the TP Chern numbers of all qps are known, the Chern number of the incompressible groundstate can be derived from equation (14),


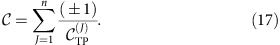


Here (−1) needs to be inserted if the elementary qh excitation of flavour *J* is used, with 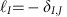 and (+1) for elementary qps with 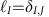.

In the main text we discuss the case of *ν*=1/*m* Laughlin states where *n*=*t*=1 and *K*=*m*. In this case there exists one qh type, with fractional charge e*=−*e*/*m*. According to equation (16) the Chern number of the TP consisting of an impurity bound to a hole is given by 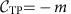. We confirm this by a microscopic calculation for a fractional Chern insulator in Fig. 3. Then, equation (17) predicts a fractional Chern number 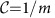 of the incompressible Laughlin state, in agreement with the established result by Niu *et al.*^5^

### Exact polaron transformation

To calculate the topological invariants characterizing TPs exactly, we need its full wavefunction 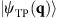 for any given value of the total TP quasimomentum **q**. Here we consider lattice systems for which we develop a method allowing to calculate 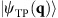 using exact numerical methods. Our approach is based on the LLP unitary transformation^50^ introduced in the context of conventional polaron physics, which makes the conservation of the polaron momentum explicit. The effect of the external force **F** acting on the impurity is also discussed in this framework.

Our starting point is the impurity-centred LLP transformation


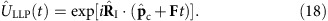


To define the impurity position operator 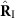 and the fermion momentum operator 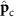, a gauge choice is made. We introduce the magnetic unit cell of size *a*_*x*_ × *a*_*y*_ for the fermion Hamiltonian 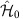, see equation (9), which contains an integer number of flux quanta. Using the Landau gauge as in equation (9) and assuming *α*=*r*/*s* with *r*, *s* integers, we have *a*_*x*_=*a* and *a*_*y*_=*sa*. Next, we label sites within the unit cell by an integer *μ* and define the impurity position operator


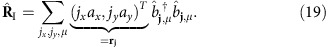


Here the integers *j*_*x*,*y*_ label unit cells, and (**j**, *μ*) is merely an alternative way of parametrizing the site indices (*m*, *n*) that were used previously in the definition of the model. Hence, we see that 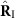 represents only the position of the unit cell, but not the positions of individual sites *within* one cell. Similarly, the fermion momentum operator is


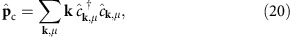


where we introduced operators in momentum space 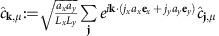. The wavevector **k** takes quantized values **k**=2*π*(*i*_*x*_/*L*_*x*_, *i*_*y*_/*L*_*y*_)^*T*^ for integers *i*_*x*_=1, ..., *L*_*x*_/*a*_*x*_ and *i*_*y*_=1, ...., *L*_*y*_/*a*_*y*_ and with *L*_*x*,*y*_ denoting system size in *x* and *y* directions. Note that, although the impurity lattice has a smaller period of *a*, we have chosen the larger magnetic unit cell of the fermion model in equation (19). This is necessary to distinguish between inequivalent sites *μ* within one magnetic unit cell for both the fermions and the impurity.

We proceed by applying the LLP transformation defined above to the Hamiltonian 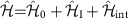 (equations (9) and (10)). The new effective Hamiltonian in the polaron frame reads 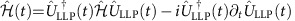. First we note that the many-body (fermion) Hamiltonian 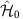 trivially commutes with the LLP transformation because of its translational invariance by multiples of one magnetic unit cell. The potential term 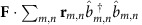 in equation (10) also commutes with the LLP transformation, as can easily be checked.

To transform the kinetic energy of the impurity, we introduce the single-particle band Hamiltonian 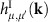 defined in the magnetic unit cell of the fermions. This allows us to write 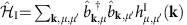 in the absence of the force **F**. The momentum operators 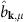 are defined as in the case of fermions 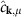 discussed above. The impurity position operator 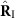 is the infinitesimal generator of displacements in quasimomentum space. Therefore, it holds


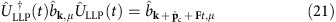


as can also be checked directly from the definition of the impurity momentum operators 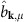. Hence, after the LLP transformation we obtain the effective impurity Hamiltonian


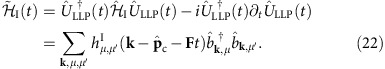


Finally, we apply the LLP transformation to the impurity–fermion interaction 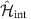. To keep the discussion general, we consider a density–density interaction of the form


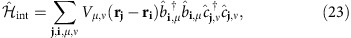


where *V*_*μ*,*ν*_(**r**) denotes an arbitrary potential. Because the LLP transformation displaces the many-body system (recall that 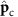 is the infinitesimal generator of fermion translations), it holds


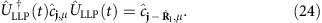


Using this relation and restricting ourselves to the subspace of one impurity, we obtain


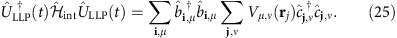


This corresponds to a static potential for the many-body fermion system, centred around the central unit cell (where **r**_**j**_=0). For the local interaction assumed in the main text it holds *V*_*μ*,*ν*_(**r**)=*Vδ*_**r,0**_*δ*_*μ*,*ν*_.

For a single impurity, the resulting Hamiltonian in the polaron frame (written in second quantization for notational convenience) is


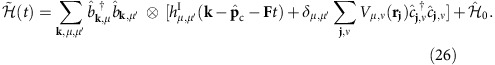


Here we used the relation 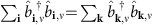 to make the conservation of the TP momentum apparent, 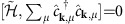. Equation (26) demonstrates that the force **F** directly couples to the TP momentum. Using exact diagonalization techniques we solved the TP band Hamiltonian equation (26) for different values of **k** (Figs 2 and 3). This allows us to extract the TP Chern number 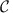 for systems with a small number of fermions.

### Fractional TP Chern numbers

Now we discuss TPs with qp charge e*/*e*=*p*/*q*, which is not the inverse of an integer, *p*≠1, assuming *p*, *q* are relative prime. In this case, the topological field theory of the TP presented above predicts a Chern number


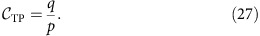


Because the TP Chern number needs to be an integer because of gauge invariance, the groundstate manifold has to be at least *p*-fold degenerate. In that case all states share a total Chern number 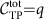.

To understand the origin of this degeneracy we investigate the *ν*=1/3 Laughlin state. To this end we bind *p*=2 qhs to the impurity, which realizes the simplest non-trivial fraction e*/*e*=−2/3 because each of the qhs has charge e*/*e*=−1/3. Every qh sees the original host particles as a source of magnetic flux. In a mean-field theory this leads to a homogeneous effective magnetic field 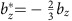 seen by the impurity.

Our reasoning so far relies on the effective low-energy topological field theory, which does not take into account lattice effects. Let us now treat the lattice as a perturbation, which is justified when the magnetic flux per plaquette 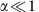 is sufficiently small. The lattice defines the magnetic unit cell of the TP, the size of which, 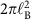, is equal to that of the original magnetic unit cell of the host particles. The size of the magnetic unit cell in the effective field 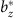, on the other hand, is larger by a factor of 3/2, 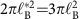 (Supplementary Fig. 1). To construct groundstates in the full lattice model from the groundstate in the effective continuum model, we choose the smallest commensurable unit cell, with a size 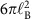.

In reciprocal space, we thus arrive at a description of the system in a reduced zone with size 1/3 of the original magnetic BZ. Because it has half the size of the effective magnetic BZ, it contains two degenerate groundstates. (As a result of the lattice, small gaps may open in some places in the BZ.) We can thus understand the twofold degeneracy as a consequence of the incommensurability of the original magnetic unit cell and the effective magnetic unit cell seen by the impurity.

We used our numerics to verify the existence of a twofold degenerate TP band in small systems (Supplementary Fig. 2a). The corresponding Berry curvature is compatible with a total Chern number 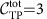 shared by the two degenerate states (Supplementary Fig. 2b). Details of our calculations are discussed in Supplementary Note 1.

### Data availability

The data that support the findings of this study are available from the corresponding author upon request.

## Additional information

**How to cite this article:** Grusdt, F. *et al.* Interferometric measurements of many-body topological invariants using mobile impurities. *Nat. Commun.* 7:11994 doi: 10.1038/ncomms11994 (2016).

## Supplementary Material

Supplementary Information Supplementary Figures 1-2, Supplementary Note 1 and Supplementary References
