# Supplementary material for: Interferometric measurements of many-body topological invariants using mobile impurities
Source: Nat Commun. 2016 Jun 17;7:11994. doi: 10.1038/ncomms11994 (PMC4915020; doi:10.1038/ncomms11994)
Supplement: Supplementary Information — Supplementary Figures 1-2, Supplementary Note 1 and Supplementary References [file ncomms11994-s1.pdf]

Supplementary Figures

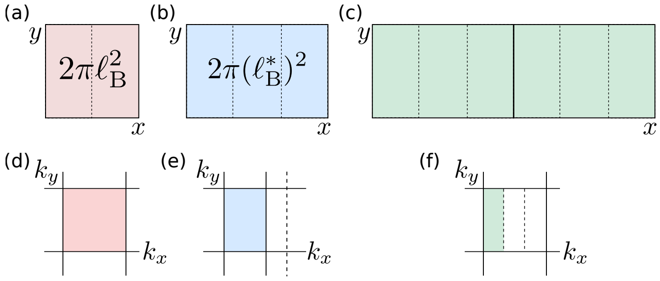

Supplementary Figure 1. **Incommensurate unit cells** The size of the magnetic unit cell of host atoms (a) is incommensurable with the size of the effective magnetic unit-cell (b) seen by the impurity bound to two fractionally charged quasiparticles. The smallest commensurable unit cell is shown in (c). In (d)-(f) the corresponding magnetic BZs are sketched. We consider the case of a $\nu = 1/3$ Laughlin state with two quasiholes (charges $e^*/e = -1/3$) bound to the impurity.

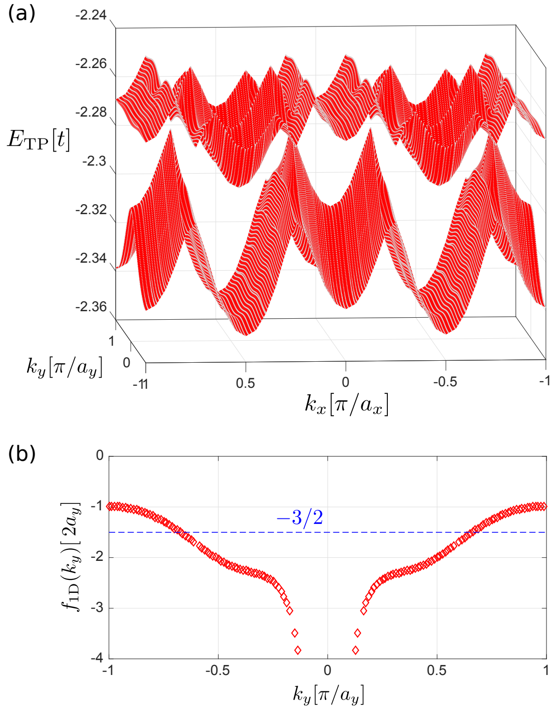

Supplementary Figure 2. **TP bandstructure and Berry curvature** The lowest four bands of the TP bandstructure are shown in (a) for a case where two quasiholes with charges $e^*/e = 1/3$ are bound to the impurity. The bands come in degenerate pairs (not shown explicitly). The integrated Berry curvature $f_{1D}(k_y)$, see Eq.(SM1), is shown in (b) for the two degenerate TP bands. The result is compared to the constant value $-3/2$ expected from the low-energy topological field theory. The model used here is described in Supplementary note 1. We simulated $N = 2$ fermions on a 8×4 lattice with $\alpha = 1/4$. This corresponds to $N_\phi = 8$ flux quanta in the system. The impurity hopping is $J = 0.1t$ and the interaction strengths are $U = 10t$ and $V = 0.5t$.

Supplementary Note 1 To check our prediction of a two-fold degenerate ground state numerically, we simulated a fractional Chern insulator at filing $\nu = 1/3$ with $N = 2$ fermions and with two qh excitations. To make our numerics more robust, we implemented the Kapit-Mueller lattice model [1], where, instead of the nearest neighbor hoppings from Eq.(9) in the main text, long range tunnelings as suggested in Ref.[1] are used. This leads to flat bands, reducing the quasiparticle dispersion in the TP Hamiltonian. The impurity Hamiltonian, see Eq.(10) in the main text, is unchanged. To trap two quasiholes efficiently, we added additional nearest neighbor interactions of strength $V/2$ to the local interaction $\hat{\mathcal{H}}_{\rm int}$ below Eq.(10) in the main text.

We find that the resulting TP bandstructure has a numerically exact two-fold groundstate degeneracy. Also the higher bands are two-fold degenerate. This is in agreement with our theoretical prediction. The lowest bands of the TP bandstructure are shown in Supp.Fig.2 (a). We observe additional degeneracies between the bands, found on the axis $k_y = 0$. This suggests that the microscopic binding of the quasiholes to the impurity breaks down at these values of TP momenta. We think that this is a finite size effect, and the microscopic binding of qhs to the impurity deserves a more careful analysis which will be devoted to future work.

We also calculated the Berry curvature ${\rm tr}\mathcal{F}(\boldsymbol{k})$ of the TP, where the trace is over the two degenerate states. In Supp.Fig.2 (b) we plot the integrated curvature along x-direction,

$$f_{1D}(k_y) = \int_{-\pi/a_x}^{\pi/a_x} dk_x {\rm tr}\mathcal{F}(\boldsymbol{k}). \qquad \text{(SM1)}$$

We compare it to the value $f_{1D}^0 = -3 \times 2a_y$ expected for a homogeneous Berry curvature which gives rise to the expected Chern number $\mathcal{C}_{\rm TP}^{\rm tot} = -3$. Away from $k_y = 0$ the average Berry curvature agrees with the predicted value f_{1D}^0. Around $k_y = 0$, on the other hand, the Berry curvature becomes large, which we attribute to an effect of the additional degeneracies with higher bands identified in Supp.Fig.2.

Supplementary references

[1] E. Kapit and E. Mueller, "Exact parent Hamiltonian for the quantum Hall states in a lattice," Physical Review Letters **105**, 215303 (2010).
